# Supplementary material for: Prevalence of metabolic syndrome and its association with cardiovascular disease risk and common risk factors amongst healthcare workers in Pakistan
Source: PLOS Glob Public Health. 2025 Mar 3;5(3):e0004135. doi: 10.1371/journal.pgph.0004135 (PMC11875290; doi:10.1371/journal.pgph.0004135)
Supplement: Attachment — Submitted filename: Response to Reviewers.docx [file pgph.0004135.s002.docx]

To,

The Editor Reviewers,

PLOS Global Public Health

Date: November 16, 2024

**Subject: Addressing Editor/Reviewer Comments for PGPH-D-24-01994**

Dear Editor,

We sincerely appreciate the thoughtful feedback from the reviewers and have addressed the concerns raised by them. We believe that the reviews have made the manuscript stronger. The detailed response to each comment is listed below.

Thank you again for your guidance.

Kind regards,

Authors of PGPH-D-24-01994

PGPH-D-24-01994

Prevalence of metabolic syndrome and its association with cardiovascular disease risk and common risk factors amongst healthcare workers in Pakistan
PLOS Global Public Health

Dear Dr. Khan,

Thank you for submitting your manuscript to PLOS Global Public Health. After careful consideration, we feel that it has merit but does not fully meet PLOS Global Public Health’s publication criteria as it currently stands. Therefore, we invite you to submit a revised version of the manuscript that addresses the points raised during the review process.

As outlined by Reviewer-1 both FRS and MetS are indicators of CVD risk. As such, the direction of this study and its contribution to the literature remain ambiguous. Please carefully address this concern (along with other reviewer comments) and revise the manuscript accordingly including the study aims if required.

Please submit your revised manuscript by Dec 02 2024 11:59PM. If you will need more time than this to complete your revisions, please reply to this message or contact the journal office at globalpubhealth@plos.org. When you're ready to submit your revision, log on to https://www.editorialmanager.com/pgph/ and select the 'Submissions Needing Revision' folder to locate your manuscript file.

Please include the following items when submitting your revised manuscript:

- A rebuttal letter that responds to each point raised by the editor and reviewer(s). You should upload this letter as a separate file labeled 'Response to Reviewers'.
  - Included
- A marked-up copy of your manuscript that highlights changes made to the original version. You should upload this as a separate file labeled 'Revised Manuscript with Track Changes'.
  - Included
- An unmarked version of your revised paper without tracked changes. You should upload this as a separate file labeled 'Manuscript'.
  - Included

Guidelines for resubmitting your figure files are available below the reviewer comments at the end of this letter.

We look forward to receiving your revised manuscript.

Kind regards,

Biplab Datta, Ph.D.
Academic Editor
PLOS Global Public Health

Journal Requirements:

Additional Editor Comments (if provided):


[Note: HTML markup is below. Please do not edit.]

Reviewers' comments:

Reviewer's Responses to Questions

**Comments to the Author**

1. Does this manuscript meet PLOS Global Public Health’s [publication criteria](https://c05y1x9s.r.us-east-2.awstrack.me/L0/https:%2F%2Fjournals.plos.org%2Fglobalpublichealth%2Fs%2Fcriteria-for-publication/1/010f0192a11b531f-1a5420c5-e2d0-4cdf-aa48-82d46bd9c2d3-000000/MuV0mXQsoJm2o133tkI6OxMMeEg=181)? Is the manuscript technically sound, and do the data support the conclusions? The manuscript must describe methodologically and ethically rigorous research with conclusions that are appropriately drawn based on the data presented.

Reviewer #1: Partly

Reviewer #2: Yes

2. Has the statistical analysis been performed appropriately and rigorously?

Reviewer #1: I don't know

Reviewer #2: Yes

3. Have the authors made all data underlying the findings in their manuscript fully available (please refer to the Data Availability Statement at the start of the manuscript PDF file)?

The [PLOS Data policy](https://c05y1x9s.r.us-east-2.awstrack.me/L0/https:%2F%2Fjournals.plos.org%2Fglobalpublichealth%2Fs%2Fdata-availability/1/010f0192a11b531f-1a5420c5-e2d0-4cdf-aa48-82d46bd9c2d3-000000/f-DKteuKLVvFzptyq7SVSbglVIs=181) requires authors to make all data underlying the findings described in their manuscript fully available without restriction, with rare exception. The data should be provided as part of the manuscript or its supporting information, or deposited to a public repository. For example, in addition to summary statistics, the data points behind means, medians and variance measures should be available. If there are restrictions on publicly sharing data—e.g. participant privacy or use of data from a third party—those must be specified.

Reviewer #1: Yes

Reviewer #2: Yes

4. Is the manuscript presented in an intelligible fashion and written in standard English?

PLOS Global Public Health does not copyedit accepted manuscripts, so the language in submitted articles must be clear, correct, and unambiguous. Any typographical or grammatical errors should be corrected at revision, so please note any specific errors here.

Reviewer #1: Yes

Reviewer #2: Yes

5. Review Comments to the Author

Please use the space provided to explain your answers to the questions above. You may also include additional comments for the author, including concerns about dual publication, research ethics, or publication ethics. (Please upload your review as an attachment if it exceeds 20,000 characters)

Reviewer #1: Thank you for the opportunity to review this manuscript. The aim of the study, which is to analyze the cardiovascular disease (CVD) risk among healthcare workers (HCWs), is a valuable addition to the literature. Below are my major and minor comments on the manuscript:

We thank the reviewer for the encouraging comments.

Major Comment: The study provides important insights by analyzing the CVD risks among HCWs and comparing them with overall population outcomes from the literature. However, it is unclear why the authors analyzed the association between the Framingham Risk Score (FRS) and Metabolic Syndrome (MetS) among HCWs. Literature (Wannamethee SG, Shaper AG, Lennon L, Morris RW. Metabolic syndrome vs Framingham Risk Score for prediction of coronary heart disease, stroke, and type 2 diabetes mellitus. Archives of Internal Medicine. 2005 Dec 12;165(22):2644-50.) indicates that both FRS and MetS are calculators of CVD risks. The authors need to discuss their hypothesis about how one determines the other and describe the objective behind using these two indicators as the dependent and independent variables in the logistic regression model.

In existing literature, both FRS and MetS are recognized as CVD risk predictors; however, only FRS has been validated with clinical outcomes in the South Asian population. ^(Garg N, Muduli SK, Kapoor A, et al. Comparison of different cardiovascular risk score calculators for cardiovascular risk prediction and guideline recommended statin uses. Indian heart journal. 2017;69(4):458-63).^ Therefore, we assessed the predictability of MetS based on FRS, as a tool for predicting the risk of CVD in Pakistani population of HCWs as MetS is yet to be validated with clinical outcomes in South Asians. Additionally, given that the association between MetS components and CVD risk varies across populations, we examined which of the five MetS components most strongly correlates with high CVD risk, using FRS as a surrogate measure.

We have duly incorporated this rationale in our manuscript in the background, methods and discussion sections.

Minor Comment: One of the limitations mentioned in the manuscript pertains to the generalizability of the results. The statement in lines 40-41 of the abstract should be edited to better reflect this limitation.

The abstract has been modified to incorporate this important limitation. The revised sentence is as follows, “While our results may not be generalizable to HCWs across South Asia, we observed a high prevalence of MetS among HCWs in our study. “

Reviewer #2:

1. The conclusion in the abstract is too lengthy.

Thank you for pointing this out. The conclusion has been revised ensuring brevity while incorporating the key finding.

2. Multivariate logistic regression table is showing only model 3. why not model 1 and 2.

The logistic regression model included all known CVD risk factors that were available. The methods section has been amended to show this.

3. Add updated literature in references. There is no study from 2024.

We have now incorporated studies from 2024 in our manuscript as per suggestion.

4. The study has reported the MetS is higher (37%) in HCWs compared to the general population (28%). However, the reason for this higher frequency of HCW is not reported. The study could be improved by identifying/reporting the possible reason for this higher percentage in HCWs compared to the general population.  Secondly, HCWs include doctors, nurses and paramedics. Which particular HCWs are more at risk should be highlighted if this information is not available, it should be highlighted in the limitation of the discussion section.

We conducted a cross-sectional study aiming to understand the prevalence of MetS and association with CVD risk in HCWs. We did not aim to investigate the underlying causes, and hence have not added hypotheses to the discussion. Future prospective research may be able to explore these.

As we had de-identified data, we are unable to comment on the HCW cadres or the region from which the HCWs hailed from. This has now been incorporated as a limitation in our study.

5. A considerably higher elevated waist circumference, overweight and obesity along with elevated low HDL are very strong findings. Particularly again in the HCWs this is a public health concern and should be reported.

South Asians are known to have higher waist circumferences compared to other ethnic groups. Elevated waist circumference and obesity are linked with low HDL levels as published by ^(Stadler JT, Lackner S et al. Obesity affects HDL metabolism, composition and subclass distribution. Biomedicines. 2021 Feb 27;9(3):242) and (Cong X, Liu S, Wang W, Ma J, Li J. Combined consideration of body mass index and waist circumference identifies obesity patterns associated with risk of stroke in a Chinese prospective cohort study. BMC public health. 2022 Feb 18;22(1):347).^ These metabolic abnormalities collectively increase the risk of CVD. Moreover, studies have consistently shown that HCWs face a heightened risk of CVD compared to age-matched individuals in the general population. ^(Joseph B, Joseph M. The health of the healthcare workers. Medknow; 2016. p. 71-2.) (Dele-Ojo BF, Raimi TH, Fadare JO, Dada SA, Ajayi EA, Ajayi DD, et al. Association between metabolic syndrome and healthcare work status in Ekiti State, Nigeria. Pan African Medical Journal. 2021;39(1)).^ Given this common understanding, we believe it may not be necessary to emphasize this as a distinct public health concern in our report.

We have now incorporated this in our discussion.

6. The study states that HCWs from all over Pakistan are included in the data set. However, there is no information available about the pattern of the disease distribution with respect to province or city as well.

As the data from HCWs was de-identified, we are unable to comment on distribution of MetS according to province. We have now incorporated this in our limitation.

6. PLOS authors have the option to publish the peer review history of their article ([what does this mean?](https://c05y1x9s.r.us-east-2.awstrack.me/L0/https:%2F%2Fjournals.plos.org%2Fglobalpublichealth%2Fs%2Feditorial-and-peer-review-process%23loc-peer-review-history/1/010f0192a11b531f-1a5420c5-e2d0-4cdf-aa48-82d46bd9c2d3-000000/x9pkXkf2eyz54D9MdtjVyAAWZIE=181)). If published, this will include your full peer review and any attached files.


**Do you want your identity to be public for this peer review?** If you choose “no”, your identity will remain anonymous but your review may still be made public.


For information about this choice, including consent withdrawal, please see our [Privacy Policy](https://c05y1x9s.r.us-east-2.awstrack.me/L0/https:%2F%2Fwww.plos.org%2Fprivacy-policy/1/010f0192a11b531f-1a5420c5-e2d0-4cdf-aa48-82d46bd9c2d3-000000/ofW0LfIuhzDTXvRwOJOAZGiXotw=181).

Reviewer #1: No

Reviewer #2: **Yes:** Syed Omair Adil

[NOTE: If reviewer comments were submitted as an attachment file, they will be attached to this email and accessible via the submission site. Please log into your account, locate the manuscript record, and check for the action link "View Attachments". If this link does not appear, there are no attachment files.]

While revising your submission, please upload your figure files to the Preflight Analysis and Conversion Engine (PACE) digital diagnostic tool, https://pacev2.apexcovantage.com/. PACE helps ensure that figures meet PLOS requirements. To use PACE, you must first register as a user. Registration is free. Then, login and navigate to the UPLOAD tab, where you will find detailed instructions on how to use the tool. If you encounter any issues or have any questions when using PACE, please email PLOS at figures@plos.org. Please note that Supporting Information files do not need this step.
